# Evaluating the Impact of Different Natural History Modeling Methods on Cost-Effectiveness Decisions: A Case Study in Duchenne Muscular Dystrophy

**DOI:** 10.1177/23814683261447231

**Published:** 2026-06-11

**Authors:** Jonathan Broomfield, Keith R. Abrams, Michael J. Crowther, Michela Guglieri, Nicholas R Latimer, Mark J. Rutherford

**Affiliations:** Biostatistics Research Group, Department of Population Health Sciences, University of Leicester, Leicester, UK; Department of Respiratory Sciences, University of Leicester, Leicester, UK; Department of Statistics & Warwick Medical School, University of Warwick, Coventry, UK; Centre for Health Economics, University of York, York, UK; Red Door Analytics, Stockholm, Sweden; John Walton Muscular Dystrophy Research Centre, Newcastle University and Newcastle Hospitals NHS Foundation Trust, Newcastle, UK; School of Health and Related Research (ScHARR), University of Sheffield, Sheffield, UK; Biostatistics Research Group, Department of Population Health Sciences, University of Leicester, Leicester, UK

**Keywords:** rare diseases, economic evaluation, Duchenne muscular dystrophy

## Abstract

**Introduction.** Cost-effectiveness analyses are vital in guiding decisions on treatment reimbursement. Natural history models are central to these, enabling the estimation of long-term costs and quality-adjusted life-years (QALYs) in the absence of lifetime trial data. Rare disease data are often scarce, resulting in disease progression being estimated through clinical assumptions. This study aims to evaluate how different modeling approaches influence cost-effectiveness estimates in rare disease health technology assessments (HTAs), using Duchenne muscular dystrophy (DMD) as a case study. **Methods.** A published economic model was used to compare 2 approaches for estimating disease progression: an assumption-based method relying on clinical plausibility and data-driven methods using data from 1,005 patients with DMD across 8 studies. Transition probabilities were estimated assuming increasing flexibility of study heterogeneity and compared with a simulated treatment cohort. Models were evaluated by comparing incremental cost-effectiveness ratios (ICERs) across approaches. No gold standard exists, so the plausibility of predictions was evaluated by comparing survival and disease progression estimates to published milestones. **Results.** Results showed that although the assumption-based model was clinically plausible, it predicted higher QALY gains (0.77) and lower ICERs (£1.96M per QALY) than data-driven methods did, which estimated QALY gains of 0.25, 0.26, 0.27, and 0.28 and ICERs of £6.2M, £6.2M, £5.8M, and £5.7M per QALY for the least to most flexible models, respectively. **Limitations.** No covariate effects or updated cost and utility data were incorporated, as the study purpose was a methodological comparison between approaches. Analyses were deterministic not probabilistic. **Conclusions and Implications.** This study emphasizes the critical role of model selection for HTA in rare diseases, showing that cost-effectiveness estimates from robust data-driven approaches can differ from clinically plausible assumption-based models.

**Highlights:**

- The choice of a natural history modeling method can drastically alter the cost-effectiveness results in rare disease evaluations.
- A case study in Duchenne muscular dystrophy demonstrates how different modeling approaches yield divergent cost-effectiveness outcomes.
- Assumption-based models, even when clinically plausible, may underestimate measures of cost-effectiveness and result in less reliable guidance for decision makers.
- Data-driven models using real-world patient data provide more reliable estimates for health technology assessment (HTA).
- This study offers practical guidance for analysts and HTA bodies on selecting robust modeling approaches in rare disease contexts.

## Graphical abstract

**Visual Abstract:**
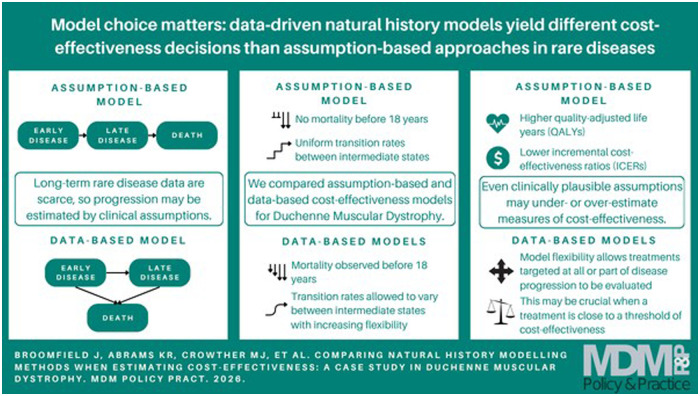
This is a visual representation of the abstract.

## Introduction

Cost-effectiveness analyses (CEAs) are a cornerstone of health technology assessment (HTA), informing decisions about the reimbursement of new treatments and therapies. Central to a CEA is the appropriate modeling of disease progression in the absence of lifetime clinical trial data. Natural history models (NHMs) enable the estimation of long-term costs and quality-adjusted life-years (QALYs) by predicting how patients move through health states over time. These are especially important when treatment effects extend beyond the duration of clinical trials. Data-driven approaches are likely to lead to more reliable estimation, but for rare disease CEAs, access to natural history data can be problematic,^1^ meaning some models instead rely on clinical assumptions to estimate disease progression. Even with data access, standard statistical methods may not be applicable due to the scarcity and heterogeneity inherent in rare disease data.^2^

In the United Kingdom, the National Institute for Health and Care Excellence (NICE) employs specific thresholds, typically between £20,000 and £30,000 per QALY,^3^ to determine whether a new technology offers sufficient value for money. However, for rare diseases, these thresholds are often less stringent^4^ due to the higher costs associated with treatment development and acquisition and the limited evidence base inherent to such conditions.^5^ The highly specialized technology (HST) evaluation route allows for higher cost-effectiveness thresholds of £100,000 to £300,000 per QALY in exceptional cases.^6^ The upper limit is reserved for significant QALY gains of up to 30 QALYs or more,^7^ which is a very high threshold to meet. To be classed as an HST, the disease must be rare, with a very small number of patients eligible for treatment, no alternative approved treatment, and a shortened life expectancy.^6^ NICE are currently reviewing the HST criteria.^8^

Duchenne muscular dystrophy (DMD) is a rare neuromuscular disease that nearly exclusively affects males, with a global incidence of 1 in 3,600 to 6,000 live male births^9^ and a UK prevalence of about 2,500.^10^ Ataluren received NICE approval for the treatment of DMD in 2014 through HST3^11^ and HST22.^12^ Previous research has compared different statistical modeling approaches to a NHM for DMD.^13^ An economic model framework for DMD has also been published,^14^ although this is solely reliant on (biologically plausible) clinical assumptions to model disease progression. The original aim of the study was to describe different cost-effectiveness models based on varying measures of disease progression of patients.^14^ The DMD Functional Ability Self-Assessment Tool (DMDSAT)^15^ was compared with commonly used model structures based on ambulatory status and ventilation support.

Building on this work, this study focuses on DMD as a case study, using the previous economic model framework based on ambulatory status to show how data-driven models can improve on assumption-based models, even when the assumptions are based on clinically plausible evidence, and assesses the impact of this on the results of a CEA. The assumption-based and data-driven approaches can yield substantially different results due to the nature of their underlying inputs. Assumption-based models rely on expert opinion and clinical plausibility, which may oversimplify disease progression and omit variability observed in real-world data. In contrast, data-driven models incorporate observed patient-level transitions, capturing heterogeneity and potentially more accurate estimates of disease progression. These differences can significantly affect cost-effectiveness outcomes, particularly in rare diseases where small changes in assumptions or data can lead to large shifts in incremental cost-effectiveness ratios (ICERs). This distinction is central to the contribution of this article. While data-driven approaches are expected to provide more robust and credible estimates, consideration is required of the reasons behind deviations between differences (both between data-driven methods and when compared with assumption-based approaches). Unlike prior studies that focused on either model development or economic evaluation in isolation, this analysis integrates both elements to highlight methodological implications for HTA. In addition, the potential for testing and identifying cost-effective treatments targeted at particular stages of a disease (rather than across the whole NHM) is explored. Sensitivity analyses explore situations in which the model choice is most pertinent and how cost-effective thresholds might be reached in rare disease HTA.

This study aims to evaluate how different natural history modeling approaches influence cost-effectiveness estimates in the context of rare diseases, using DMD as a case study. Specifically, we compare assumption-based and data-driven methods for estimating disease progression and assess their impact on ICERs. The findings are intended to inform health economists, analysts, and decision makers involved in HTA, particularly in rare disease contexts in which data limitations often necessitate methodological compromises. By highlighting the implications of model choice, this article provides practical guidance for selecting robust modeling approaches in future economic evaluations.

## Methods

This study evaluates the impact of different natural history modeling approaches on cost-effectiveness estimates. It focuses on a population of patients with DMD, beginning with diagnosis at age 5 y. The intervention is a hypothetical treatment assumed to reduce disease progression rates by 25% and cost £100,000 per patient per year, compared against a standard-of-care cohort based on either historical disease progression data or clinically plausible assumptions. The outcomes of interest are lifetime costs, QALYs and ICERs.

### Economic Model Framework

**Figure 1:**
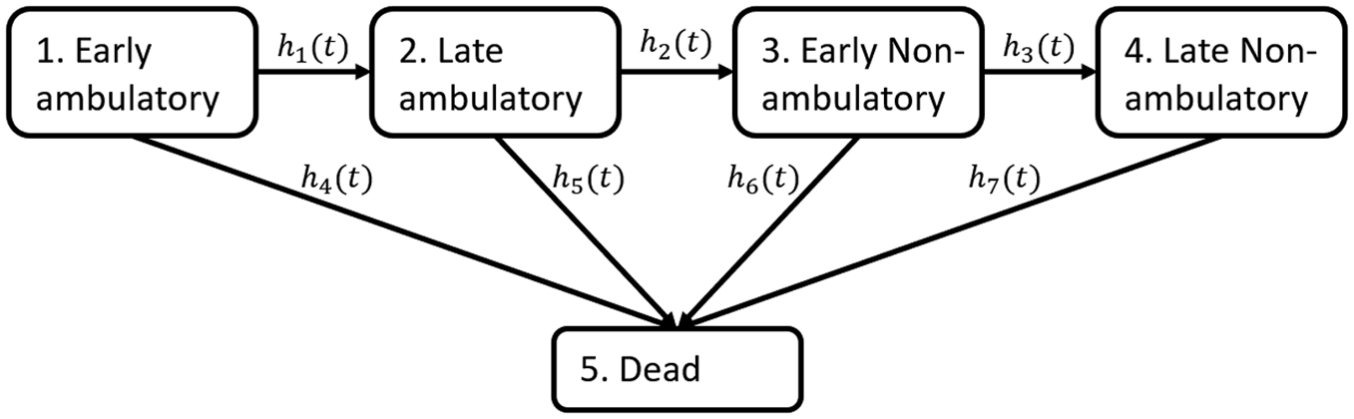
Natural history model structure based on ambulatory status, with hazard function *h_k* (*t*) on transition *k*.

To highlight differences in natural history modeling methods on decision models, the ambulatory status model from the published economic framework is used as a case study. This model defines 4 health states—early ambulatory (where patients are aged approximately 5–7 y), late ambulatory (aged approximately 8–11 y), early nonambulatory (aged approximately 12–15 y), and late nonambulatory (aged approximately 16 y and older)—with mortality as the absorbing state (Figure 1).

Due to the rarity of DMD, disease progression data were unavailable to the authors publishing the economic model framework, in particular for the newly developed DMDSAT.^14^ Transition probabilities between states were instead estimated through a number of clinically justified assumptions identified from a targeted literature review. Patients in the economic model framework were assumed to enter the early ambulatory state at age 5 y, since this is the average age of diagnosis,^16^ and progress through subsequent states to reflect increasing disease severity. As such, no backwards transitions were allowed. Mortality was allowed from all disease states, and an increasing hazard of death as disease severity increases was assumed.^17^ Specifically, patients were assumed to spend on average 4 y in each of the intermediate states,^18^ corresponding to a constant annual transition probability of 0.159 for hazards $h_{1}$ to $h_{3}$ . A median survival of 25 was assumed,^19^ leading to constant annual transition probabilities of 0.094 for hazards $h_{4}$ to $h_{7}$ . There was no assumed mortality between the ages of 5 and 18 y, meaning the annual transition probabilities for these hazards were 0 for the first 13 y of the model. The mortality probabilities were also assumed to increase by 15% each year beyond the age of 35 y, so that no patients were observed to survive beyond 50 y in the standard-of-care cohort. These transitions were assumed to be consistent, with no variation across the study population.

In this study, the standard-of-care cohort simulation was replicated under these disease progression assumptions. To verify the accuracy of this replication, a comparison of the disease progression estimates (presented as transition probabilities) was conducted. For additional accuracy, the plots were uploaded to WebPlotDigitizer,^20^ and transition probabilities at a range of ages were compared.

The economic model adopted a UK health care perspective, incorporating direct medical costs and quality-of-life estimates for each health state. Annual costs and utilities associated with being in the 4 intermediate health states that were calculated by Landfeldt et al.^14^ are shown in Table 1. The costs shown are direct medical costs, that is, from the health care perspective, and utilities are for patients (rather than caregivers).

**Table 1:** Annual Direct Medical Costs and Patient Utilities for the Duchenne Muscular Dystrophy Natural History Model Based on Ambulation

Health State	Annual Cost	Utility
Early ambulatory	£10,670	0.699
Late ambulatory	£11,190	0.607
Early nonambulatory	£16,490	0.224
Late nonambulatory	£27,590	0.146

The costs were obtained from 2012 data from the United States, exchanged to UK currency and inflated to 2015 values.^14^ The values in Table 1 have been taken directly from the original study and have not been altered or updated for this analysis, as the purpose is to compare different modeling techniques rather than to conduct a genuine CEA of a DMD health technology.

### Simulation of Treatment Effects

A hypothetical treatment cohort was simulated in the economic model framework to represent a new health technology for DMD.^14^ The treatment was assumed to cost £100,000 per patient per year (in line with some rare disease treatments at the time of the economic framework publication^21^ and approximately half the current cost of ataluren^12^). The treatment was assumed to reduce all intermediate annual transition probabilities by 25% (consistent with the observed efficacy of glucocorticoid treatment in patients with DMD^22^) and to not affect utility values. A treatment cohort was simulated in the same way in this study to enable a demonstrative CEA to be conducted. The hypothetical treatment was assumed not to affect utility values or introduce adverse effects. To verify the accuracy of the replication of treatment and standard-of-care cohorts, the estimated total costs and QALYs for each cohort were compared with the estimates of Landfeldt et al.^14^

### Disease Progression Data

As a comparison to the assumption-based approach adopted in the economic model framework, transition probabilities were also estimated from a collection of datasets on disease progression in patients with DMD. The datasets were made available from the Cooperative International Neuromuscular Research Group (CINRG)^23^ and C-PATH Duchenne Regulatory Science Consortium (D-RSC)^24^ and are described in detail in previous studies.^13,25^ To summarize, disease progression data were available from observational and natural history studies, placebo arms of clinical trials, and clinical test data. A total of 1,005 patients from 8 studies were included across a range of ages (4–34 y), study periods (2004–2018), and locations (North America, South America, Europe and, Asia) and contained patients on and not on steroids. The following variables were used to map patients to disease states: ability to stand from supine, ability to walk/run 10 m, North Star Ambulatory Assessment score,^26^ Brooke score,^27^ forced vital capacity percent predicted, and ventilation status (none/nighttime/full-time). Further details of the C-Path datasets are in the Supplementary Materials, and D-RSC data access applications can be made via https://c-path.org/tools-platforms/d-rsc-database/.

### Methods for Estimating Disease Progression

Four previously evaluated methods for combining individual patient data from multiple sources^13^ were used to calculate disease progression through transition probabilities between the states described in Figure 1. These consisted of a model that does not adjust for study source, a 1-stage frailty model, a 2-stage model assuming proportional baselines between studies, and a 2-stage model assuming stratified baselines. All the models assumed a Weibull baseline hazard function, and no patient-level covariate information was included (covariate information could be incorporated into all of these models but was not consistently available from the datasets, a common rare disease issue^1^). The 4 models are formulated as follows (where $h_{\mathrm{jk}}$ corresponds to the hazard $h_{k}$ on transition k for study j in Figure 1):

*No-adjustment model:*

$$h_{\mathrm{jk}}\left(t\right)=\lambda_{k}\gamma_{k}t^{\gamma_{k}-1}$$

*One-stage frailty model:*

$$h_{\mathrm{jk}}\left(t\right)=\lambda_{k}\gamma_{k}t^{\gamma_{k}-1}\exp\left(\alpha_{\mathrm{jk}}\right),\alpha_{\mathrm{jk}}N(0,\sigma_{k}^{2})$$

*Two-stage proportional model:*

$$h_{\mathrm{jk}}\left(t\right)=\lambda_{\mathrm{jk}}\gamma_{k}t^{\gamma_{k}-1}$$

$$\log({\hat{\lambda }}_{\mathrm{jk}})N(\log({\hat{\lambda }}_{k}),\sigma_{k}^{2})$$

*Two-stage stratified model:*

$$h_{\mathrm{jk}}\left(t\right)=\lambda_{\mathrm{jk}}\gamma_{\mathrm{jk}}t^{\gamma_{\mathrm{jk}}-1}$$

$$\left(\begin{array}{c} \log({\hat{\lambda }}_{\mathrm{jk}})\\ \log({\hat{\gamma }}_{\mathrm{jk}}) \end{array}\right)\sim N\left(\left(\begin{array}{c} 0\\ 0 \end{array}\right),\left(\begin{array}{cc} \sigma_{\lambda_{k}}^{2} & \mathrm{Cov}(\log({\hat{\lambda }}_{k}),\log({\hat{\gamma }}_{k}))\\ \mathrm{Cov}(\log({\hat{\lambda }}_{k}),\log({\hat{\gamma }}_{k})) & \sigma_{\gamma_{k}}^{2} \end{array}\right)\right)$$

The no-adjustment model assumes that the transition probabilities of moving from one state to the next are the same across all studies, meaning all studies have the same Weibull baseline hazard function for transition k . The 1-stage frailty model assumes that studies vary by an unobserved random effect $\alpha_{\mathrm{jk}}$ , meaning their baseline hazards differ by a proportional term $\exp(\alpha_{\mathrm{jk}})$ . Between-study heterogeneity is estimated in this model through $\sigma_{k}^{2}$ . The 2-stage proportional model also assumes that baseline hazards in each study are proportional but estimates $\lambda_{\mathrm{jk}}$ directly in each study before combining these estimates ${\hat{\lambda }}_{\mathrm{jk}}$ (on the log-scale) in a second stage to obtain a pooled estimate of $\lambda_{k}$ (with between-study heterogeneity again estimated through $\sigma_{k}^{2}$ ). The 2-stage stratified model allows the baseline hazards to vary beyond proportionality, estimating a separate shape and scale parameter for each study on each transition, and combines these estimates ${\hat{\lambda }}_{\mathrm{jk}}$ and ${\hat{\gamma }}_{\mathrm{jk}}$ (again on the log-scale) in a second stage through a multivariate meta-analysis^28^ to obtain pooled estimates of $\lambda_{k}$ and $\gamma_{k}$ . Between-study heterogeneity is now estimated through $\sigma_{\lambda_{k}}^{2}$ and $\sigma_{\gamma_{k}}^{2}$ .

A previous simulation study identified the 2-stage models (particularly the 2-stage stratified model) as the least biased estimators of transition probabilities among the rare disease scenarios considered, while the 1-stage frailty model showed low absolute bias conditional on convergence.^13^ The no-adjustment model was the most biased estimator of transition probabilities.

These 4 models were applied to the disease progression datasets. The datasets had previously been assigned to health states for DMD from a more granular NHM with 8 intermediate states^29^ (according to clinical and test records), published as part of Project HERCULES.^30^ Details of how patients were mapped to these states based on the available variables have been published previously.^29^ Disease progression could be estimated more precisely using the HERCULES model; however, this would have prevented a direct comparison with the assumption-based estimates of disease progression from the economic model framework and would have required additional estimates of costs and utilities for the extra health states in the HERCULES model. The 8 intermediate health states from the HERCULES model were therefore collapsed into the 4 intermediate states in the ambulatory model of Figure 1 (with clinical input) as follows. States 1 (early ambulatory) and 2 (late ambulatory) of the ambulatory model are equivalent to states 1 and 2 of the HERCULES model. Patients in states 3 to 6 of the HERCULES model were assigned to state 3 (early nonambulatory) of the ambulatory model, since these health states correspond to patients who cannot walk but have better health than patients in states 7 or 8 of the HERCULES model, who were as such assigned to state 4 (late nonambulatory). Observations from the datasets were then mapped from the HERCULES model health states to the ambulatory model health states accordingly.

The 4 methods produced 4 models estimating disease progression under standard of care based on patient data. No treatment data were available, and so disease progression under a treatment were simulated in the same way as the original economic framework to allow the CEA to be conducted.^14^

To evaluate the impact of different modeling approaches on disease progression, predicted health state occupancies and lengths of stay (LOS) were calculated for each method. These visualizations allow for direct comparison of how patients are expected to transition through disease stages under each model. The LOS estimates feed directly into the calculation of costs and QALYs, meaning differences in LOS estimation between methods will alter the corresponding ICERs.

### Cost-Effectiveness, Sensitivity, and Threshold Analyses

Once annual transition probabilities for the standard-of-care and treatment cohort had been estimated from the C-PATH data using the 4 models and from the previous assumption-based approach, the annual costs and utilities from Table 1 were applied to determine lifetime costs and QALYs. An annual discount rate of 3.5% was assumed. From these, ICERs were calculated for each method.

Sensitivity analyses were conducted to vary assumptions around treatment duration and efficacy, the discount rate, and annual costs/utilities. Scenarios reported in the original economic framework were replicated, including increasing or decreasing the discount rate, state utility values and baseline (direct [medical] costs), as well as treatment efficacy on mortality.^14^ In addition, treatment effects were assumed to affect only transitions out of one state at a time, representing a targeted treatment. The sensitivity analyses were conducted deterministically.

Finally, threshold analyses investigated what total treatment cost, annual treatment cost, and total QALY gain would be required to obtain a cost-effective ICER for each of the methods in the base-case scenario.

The primary aim of the analysis is to explore how different natural history modeling approaches influence cost-effectiveness outcomes. Rather than identifying a single superior model, the study compares 5 approaches, 1 assumption-based and 4 increasingly flexible data-based, within a consistent economic framework. While no formal gold standard exists, the plausibility of each model’s predictions is assessed against published literature and clinical expectations. This comparative approach highlights the sensitivity of cost-effectiveness results to the flexibility of modeling choices and aims to inform best practices in rare disease HTA.

## Results

### Disease Progression Estimates

**Figure 2:**
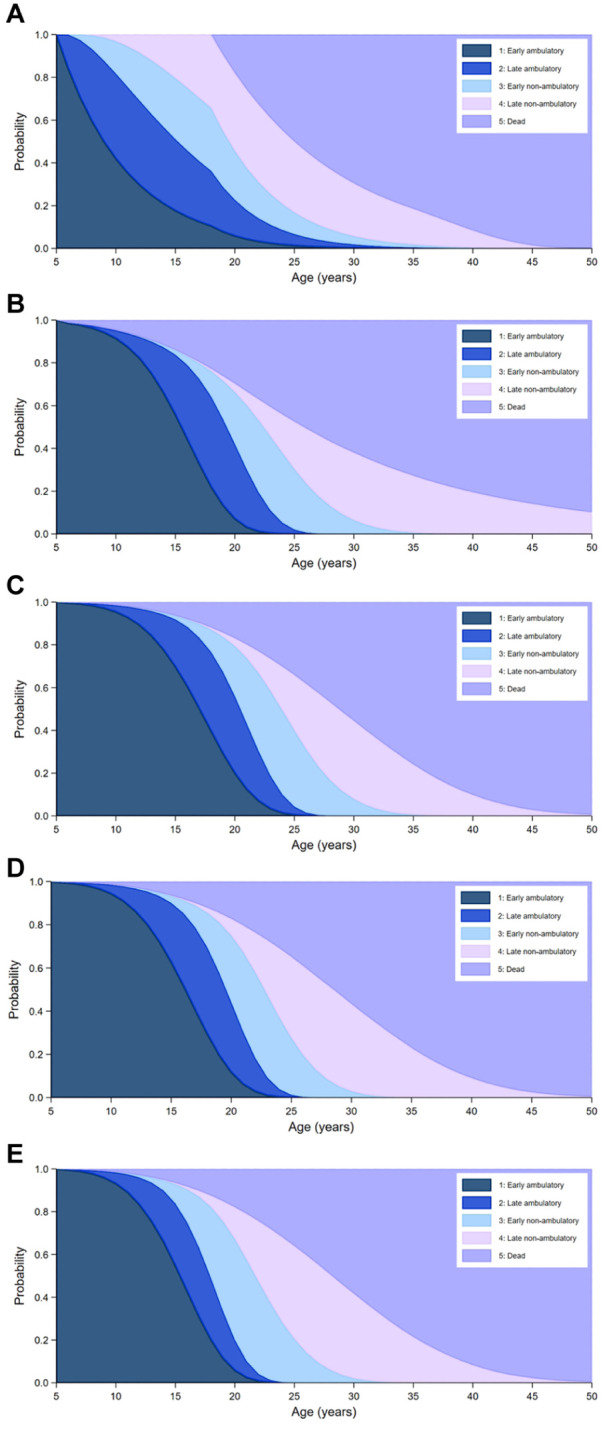
Health state occupancies after age 5 y across the ambulatory disease states from the assumption based and 4 model-based approaches for the standard of care cohort: (a) assumption-based approach,^14^ (b) no-adjustment model, (c) 1-stage frailty model, (d) 2-stage proportional model, and (e) 2-stage stratified model.

Disease progression from the 4 models is shown in Figure 2, alongside the disease progression estimated from the assumptions-based approach. Patients were assumed to start in the early ambulatory state at age 5 y. Equivalent plots for the treatment cohort are available in Figure 1 of the Supplementary Materials. Table 2 gives the total LOS in each of the intermediate states (calculated as the shaded areas in each of the stacked plots in Figure 2) alongside the median survival from each of the 5 methods. Also tabulated are estimates from the simulated treatment cohort.

**Table 2:** Mean Lengths of Stay from the Age of 5 y in the 4 Intermediate Health States and Mean/Median Survival for the 5 Methods, across the Standard-of-Care and Simulated Treatment Cohorts

Method	Assumption Based		No Adjustment		One-Stage Frailty		Two-Stage Proportional		Two-Stage Stratified	
Cohort	SoC	Trt	SoC	Trt	SoC	Trt	SoC	Trt	SoC	Trt
Early ambulatory	5.5	7.2	10.7	11.4	12.3	13.1	11.4	12.2	10.7	11.4
Late ambulatory	5.3	6.0	3.4	3.4	3.2	3.2	3.2	3.3	2.5	2.5
Early nonambulatory	4.1	4.0	3.1	3.2	3.4	3.5	3.2	3.3	3.9	4.1
Late nonambulatory	7.2	4.9	7.6	7.1	5.2	4.3	6.0	5.2	6.5	5.6
Mean survival	22.1	22.1	24.8	25.1	24.1	24.1	23.8	24.0	23.6	23.6
Median survival	25.0	25.0	25.9	26.1	28.5	28.5	28.5	28.5	28.2	28.3

SoC, standard-of-care cohort; Trt, treatment cohort.

One clear distinction between the probabilities derived from the assumption-based approach and those from the data-driven methods is the lack of mortality until age 18 y from the former; in Figure 2a, patients aged 18 y are distributed fairly evenly across the 4 intermediate health states, but no patients are predicted to die, whereas the other 4 methods predict a nonnegligible possibility of mortality from as early as age 7 y and probabilities of between 12% and 23% of mortality by age 18 y. These predictions are more closely aligned with literature estimates, which have estimated a probability of death by age 18 y of approximately 30%.^17^ The no-adjustment model estimates a 10% probability of still being alive at 50 y, which does not match clinical practice and is likely a reflection of the simpler modeling assumptions in this method. Transition probabilities from the original and reproduced assumption-based model were in agreement across a patient’s lifetime.

Patients spend longer in the early ambulatory state under the data-based models than in the assumption-based model, which could be due to different state definitions (clinical plausibility from published guidelines^18^ versus clinical test data). Patients are also distributed much more evenly across intermediate states using the assumption-based approach, which is expected since the transition probabilities were determined by assuming patients move through each state on average every 4 y. Predictions from the other models suggest patients in the C-Path datasets spend much longer in the early ambulatory state across their lifetime. There is also some variation between the predictions from the 4 models, with the no-adjustment model having much longer tails than the other 3 models. The median survival estimates from the more complex statistical models are more in line with literature estimates of median survival than from the assumption-based and no-adjustment models.^17,31^

The overall mean and median survival across the standard-of-care and treatment cohorts differs notably for only the no-adjustment methods, with a mean and median difference of 0.3 y and 0.2 y, respectively. The lack of noticeable difference in median survival between the 2 cohorts is because the treatment cohort is assumed to have no impact on mortality; in general, patients in the treatment cohort spend longer in the ambulatory states and (as a result) shorter in the nonambulatory states than patients in the standard-of-care cohort.

### CEA Results

Table 3 shows the lifetime costs and QALYs estimated for the standard-of-care and treatment cohorts, lifetime differences in costs and QALYs between the standard-of-care and (simulated) treatment cohort, and the ICER obtained from these differences for each of the 5 methods.

**Table 3:** Estimated Lifetime Costs and QALYs in the Standard-of-Care and Treatment Cohorts, Differences in Costs and QALYs between the 2 Cohorts, and ICERs Obtained from the 5 Methods

Method	Total SoC Costs	Total Trt Costs	Total SoC QALYs	Total Trt QALYs	Δ Costs (£)	Δ QALYs	ICER (£)
Assumption based	£360,000	£1,880,000	6.76	7.53	£1,520,000	0.77	£1,960,000
No adjustment	£280,000	£1,810,000	8.29	8.54	£1,530,000	0.25	£6,190,000
One-stage frailty	£310,000	£1,890,000	8.91	9.17	£1,580,000	0.26	£6,190,000
Two-stage proportional	£320,000	£1,900,000	8.89	9.16	£1,580,000	0.27	£5,820,000
Two-stage stratified	£340,000	£1,900,000	8.43	8.71	£1,560,000	0.28	£5,650,000

SoC, standard-of-care cohort; Trt, treatment cohort.

The estimates from the assumption-based model accurately replicate the estimates from the original economic framework model, which estimated a difference in costs of £1.52 million, a difference in QALYs of 0.79, and an ICER of £1.94 million. The small disparities are likely due to rounding differences in the software (Excel versus Stata) used to calculate the values.

The total difference in costs between the treatment and standard-of-care cohorts was fairly consistently estimated across all models (approximately £1.5–£1.6 million over the lifetime of a patient). This is due to the methods predicting that patients will live for approximately the same length of time; the median survival is slightly lower for the assumption-based and no-adjustment methods, leading to a slightly lower difference in costs. The differences are largely driven by the high cost of the treatment relative to the standard-of-care costs, and so the treatment cost was varied in a threshold analysis. The assumption-based approach also predicted a much higher QALY difference between the 2 cohorts (0.77 QALYs) than the other methods, all of which predicted a difference of 0.25 to 0.28 QALYs. This is mainly because the difference between the LOS for the treatment cohort and standard-of-care cohort was greater for the assumption-based method than for the data-based methods. In particular, under the assumption-based method, patients in the treatment cohort were estimated to spend 1.7 more years in the early ambulatory state and 2.3 fewer years in the late nonambulatory state than patients in the standard-of-care cohort were.

The 2-stage methods (which were previously observed to be the least biased estimators of transition probabilities in a simulation study focused on DMD disease progression^13^) gave similar estimates for the differences in costs and QALYs. The 1-stage frailty model estimated a slightly higher difference in costs, whereas the assumption-based and no-adjustment approaches gave a lower difference. Similar ICERs were obtained for the 2-stage methods (£5.7–£5.8 million per QALY), whereas the no-adjustment and 1-stage frailty models estimated higher ICERs of £6.2 million per QALY. Due to the difference in QALYs being so much higher for the assumption-based approach, the ICER from this method was vastly lower, at £2.0 million per QALY.

### Sensitivity Analysis Results

Given the disparity between the assumption-based and data-based ICER estimates, the sensitivity analyses highlighted under which scenarios these differences might be more or less apparent in this case study. None of the methods were close to achieving a cost-effective ICER, even at the increased threshold of £100,000 per QALY, due to the very high treatment cost relative to quite a low efficacy. The resulting differences in costs, QALYs, and ICERs from sensitivity analysis scenarios relating to treatment duration and efficacy are displayed in Table A1 of the Supplementary Materials.

Figure A1 in the Supplementary Materials shows the resulting differences in lifetime costs and QALYs, and corresponding ICERs, from the remaining scenarios.

Assuming that the treatment also improved survival increased QALYs and costs. In the scenarios considered, treatments that lead to higher QALY gains were less affected by modeling differences, although mortality assumptions drove the greatest variation in ICERs. Varying discount rates and baseline costs had little impact, whereas varying utility values had a high impact on ICERs, with higher utilities reducing ICERs by up to 30%. These findings again underline the finding that greater disparities between restrictive and more flexible approaches are likely to be observed when QALY gains (or treatment costs) from the standard-of-care cohort to the treatment cohort are lower.

### Threshold Analysis Results

Figure A2 in the Supplementary Materials shows the results of threshold analyses to determine the required difference in total costs, annual costs, and total QALYs, respectively, between the standard-of-care and treatment cohorts to obtain various ICERs, including a cost-effective ICER threshold of £100,000 per QALY. Threshold analyses relating to treatment cost (Figure A2a and A2b) showed clear differences between assumption-based and data-based approaches, while threshold analyses on QALYs were consistent across these (as expected, since lifetime costs were consistently estimated across models). In order to be cost-effective, the different models would require a QALY gain of between 8.2 and 8.8, a long way from the observed difference in QALYs in Table 3.

## Discussion

This study demonstrated the impact that natural history modeling approaches can have on the results of CEAs for rare diseases, using DMD as a case study. By comparing assumption-based and data-driven methods, the choice of methodology for estimating disease progression was shown to significantly influences predictions of QALYs, costs, and ICERs. A model based on ambulatory status was used due to similarities between the model structure and the available disease progression data and because of its common use in clinical practice.^18^ The disease stages are specified in published DMD clinical care guidelines to assist with diagnosis and symptom management, including neuromuscular, cardiac, psychosocial management, and many others.^18,32,33^

The assumption-based approach yielded substantially different results from the data-driven methods. It predicted longer durations in less severe health states for the treatment cohort relative to the standard-of-care cohort and perhaps most significantly assumed no mortality until the age of 18 y, leading to higher QALY differences and much lower ICERs (£1.96M per QALY versus £5.65–£6.19M per QALY for data-driven methods). This difference was driven by a much higher incremental QALY gain estimated by the assumption-based approach, with an approximately 3-fold increase in the QALY gain from this approach compared with the data-driven methods. Incremental costs were estimated relatively consistently between methods due to much higher lifetime treatment costs compared with standard-of-care costs. This finding underscores the importance of measuring both costs and QALYs alongside overall ICERs to determine what is driving differences in results between methodologies. The difference in QALYs between the assumption-based and data-based methods could be driven by assumptions around mortality, specifically, that the assumption-based model assumes no mortality until the age of 18 y. The large difference in LOS in the early ambulatory stay between the treatment and standard-of-care cohorts for this method is likely to be driven by this assumption. This may be compounded by the assumption of patients all entering the model at age 5 y, meaning differences in earlier health states of the model (corresponding to younger patients) have a greater impact on the CEA than for later health states, due to the economic discounting.

However, the optimistic outlook of the assumption-based approach is inconsistent with literature estimates and demonstrates the risks of relying on simplified, assumption-driven models. In particular, excess mortality has been observed in patients with DMD well before the age of 18 y.^17^ This study highlights that assumptions that appear clinically plausible and lead to believable transition probabilities may well be inaccurate, but this may not be obvious until a data-driven approach is applied. More thorough elicitation approaches to populate assumption-based models may lead to more similar ICER estimates with data-driven models (although a targeted literature review was conducted in the original economic model framework^14^).

While the primary conclusion that model choice significantly influences outcome estimates remains robust, we acknowledge some inconsistencies between the assumption-based and data-based models and observations in clinical practice regarding other clinical milestones beyond mortality. For instance, early ambulatory status was observed to be prolonged in the data-based models, but the median loss of ambulation was no lower than 20 y across all 5 models, much higher than has been observed in previous literature.^34^ Although the data-driven approach may still be limited by the quality and completeness of available data, it arguably offers a closer reflection of real-world disease progression than the assumption-based alternative. These limitations can be partially addressed through the application of a 2-stage stratified model, which helps mitigate bias and improve the robustness of the findings.

Among the data-driven approaches, the 2-stage stratified model offered the most flexible and robust approach to accounting for heterogeneity across data sources. The results underline the importance of accurately adjusting for study-specific differences when pooling individual patient data, particularly for rare diseases for which data are often sparse and heterogeneous. Predictions from the frailty model are taken as conditional on zero frailty, in other words, an “average” study population relative to the studies that were used to populate the NHM. A new health technology would likely necessitate a new study, separate from the populations used to estimate disease progression in the NHM. Therefore, while it is possible that this average study population represents a reasonable baseline against which to compare a new study population, it is important to evaluate where in these populations the new study sits (especially given the heterogeneity that is often present in these study populations^1^). If it is similar in nature to a more extreme population from one of the original studies, then study-specific predictions conditional on the frailty from this study would be more representative as a baseline NHM.

The costs were not altered from the conversion from 2012 US currency to 2015 UK currency, as originally published,^14^ meaning they are not representative of current medical costs in the United Kingdom. Similarly, a DMD-specific quality-of-life questionnaire, the DMD-QoL,^35^ has since been developed that may allow more accurate state utility values to be determined.

For HTA in rare diseases, where randomized controlled trial data are often unavailable,^2,36^ NHMs play a critical role in estimating disease progression and informing decision making. This study highlighted the dangers of assumption-driven methods, which can misrepresent disease progression and treatment effects, potentially compromising the robustness of cost-effectiveness estimates. Conversely, data-driven methods offer greater reliability but require careful selection of the most appropriate modeling approach based on biological plausibility, model checking, and evaluating robustness through sensitivity analysis. This is especially pertinent when it is not clear which approach is most favorable and if results from different methods vary. The methods in this study have been rigorously assessed in a rare disease setting, minimizing this risk. We recognize that many rare diseases lack the data infrastructure available for DMD, such as that provided by Project HERCULES. In such contexts, assumption-based models may be the only feasible option, and efforts should focus on improving transparency and elicitation methods to strengthen their validity.

A key limitation of this analysis is the absence of covariate effects in the NHMs. Just as the assumption-based approach oversimplified disease progression, omitting covariates may have the same impact. Incorporating patient-specific factors, such as genotype, baseline functional status, or corticosteroid use, could improve the precision of transition probability estimates and better capture variability in disease progression. This would be particularly important if costs and utilities varied substantially across patient subgroups. However, data availability often constrains the complexity of model structures (as in this case study), necessitating tradeoffs between realism and feasibility. Rare disease models, in particular, are frequently criticized for oversimplifications, such as omitting key health states. For instance, in previous NICE appraisals of DMD treatments, the exclusion of scoliosis as a health state was highlighted as a limitation.^11^ Further stratification of the ambulatory states could enhance the granularity of future models, although this would increase data requirements. More flexible model specifications for between-state transitions beyond Weibull distributions could also have been considered, although the adopted approach did offer more flexibility than previous multistate modeling of DMD, where constant transition rates between states were assumed.^14,29^

Another limitation is that analyses were deterministic rather than probabilistic. A probabilistic sensitivity analysis (PSA) could quantify uncertainty in transition probabilities, costs, and utilities and assess whether differences in ICERs between modeling approaches remain meaningful when uncertainty is considered. In this study, we did not conduct a PSA because the primary aim was methodological, comparing deterministic outputs under identical assumptions rather than providing a comprehensive cost-effectiveness evaluation and maintaining comparability with previous analysis.^14^ Future methodological work in this area would be of interest, and applications of the methods to a CEAs should incorporate a PSA to better understand the robustness of conclusions and the potential overlap in ICER distributions across methods.

The results also emphasize the value of targeting treatments to specific disease stages. Sensitivity analyses revealed that interventions focused on states with high(er) utilities, such as the early ambulatory phase of DMD, reduced ICERs more effectively than treatments applied across all health states. This targeted approach may help novel therapies achieve cost-effectiveness thresholds, particularly in the context of high costs and modest QALY gains. An important further consideration highlighted by this study is the need for robust utility data, as utility values were shown to significantly influence the outcomes in sensitivity analyses. This underscores the value of early modeling, which can help identify critical data gaps such as the need for high-quality utility estimates. Addressing these gaps may require the proactive planning of targeted studies to generate the necessary evidence.

This study was not intended as a comprehensive cost-effectiveness evaluation but rather as a methodological investigation into the effects of natural history modeling approaches on economic outcomes for a particular case study. The cost and utility estimates, derived from older data, may not reflect current clinical and economic conditions. In addition, the analyses were deterministic rather than probabilistic, limiting the evaluation of uncertainty in model inputs. Future work could incorporate probabilistic sensitivity analyses and value-of-information analyses to better quantify the uncertainty in cost-effectiveness estimates. Moreover, extending the analysis to include Bayesian frameworks may allow for more robust modeling of heterogeneity and uncertainty.^37,38^ Alternative diseases should also be explored to widen the context of the research.

### Considerations for Model Selection

Selecting an appropriate modeling approach for estimating disease progression in CEAs, particularly in rare diseases, is critical as it can substantially influence estimates. When estimating disease progression, researchers should consider the following key factors. First, what is the level of data availability and quality—are individual patient data available, and are the data heterogeneous across data sources? Second, balance clinical plausibility with statistical robustness by ensuring assumptions are evaluated and the model is externally validated where possible. Third, consider model flexibility, such as adjustments for between-study variation or covariate inclusion/stratification. Fourth, ensure the methods are reported transparently to ensure reproducibility. Finally, evaluate how sensitive the cost-effectiveness results are to model selection and assumptions. For instance, would different models lead to different reimbursement decisions?

## Conclusion

In the context of rare diseases, where robust evidence is often limited, the selection of natural history modeling methods is critical. This study emphasizes the importance of adopting advanced, data-driven approaches to accurately estimate disease progression and inform HTA decision making, highlighting the need for timely preparation of data and evidence generation plans. By demonstrating the impact of methodology on cost-effectiveness outcomes, this research provides a framework for improving the validity and reliability of economic evaluations in rare diseases, ultimately aiding in the development and reimbursement of new treatments.

## Supplemental Material

sj-docx-1-mpp-10.1177_23814683261447231 – Supplemental material for Evaluating the Impact of Different Natural History Modeling Methods on Cost-Effectiveness Decisions: A Case Study in Duchenne Muscular Dystrophy Supplemental material, sj-docx-1-mpp-10.1177_23814683261447231 for Evaluating the Impact of Different Natural History Modeling Methods on Cost-Effectiveness Decisions: A Case Study in Duchenne Muscular Dystrophy by Jonathan Broomfield, Keith R. Abrams, Michael J. Crowther, Michela Guglieri, Nicholas R Latimer and Mark J. Rutherford in MDM Policy & Practice

sj-docx-2-mpp-10.1177_23814683261447231 – Supplemental material for Evaluating the Impact of Different Natural History Modeling Methods on Cost-Effectiveness Decisions: A Case Study in Duchenne Muscular Dystrophy Supplemental material, sj-docx-2-mpp-10.1177_23814683261447231 for Evaluating the Impact of Different Natural History Modeling Methods on Cost-Effectiveness Decisions: A Case Study in Duchenne Muscular Dystrophy by Jonathan Broomfield, Keith R. Abrams, Michael J. Crowther, Michela Guglieri, Nicholas R Latimer and Mark J. Rutherford in MDM Policy & Practice

sj-docx-3-mpp-10.1177_23814683261447231 – Supplemental material for Evaluating the Impact of Different Natural History Modeling Methods on Cost-Effectiveness Decisions: A Case Study in Duchenne Muscular Dystrophy Supplemental material, sj-docx-3-mpp-10.1177_23814683261447231 for Evaluating the Impact of Different Natural History Modeling Methods on Cost-Effectiveness Decisions: A Case Study in Duchenne Muscular Dystrophy by Jonathan Broomfield, Keith R. Abrams, Michael J. Crowther, Michela Guglieri, Nicholas R Latimer and Mark J. Rutherford in MDM Policy & Practice

sj-docx-4-mpp-10.1177_23814683261447231 – Supplemental material for Evaluating the Impact of Different Natural History Modeling Methods on Cost-Effectiveness Decisions: A Case Study in Duchenne Muscular Dystrophy Supplemental material, sj-docx-4-mpp-10.1177_23814683261447231 for Evaluating the Impact of Different Natural History Modeling Methods on Cost-Effectiveness Decisions: A Case Study in Duchenne Muscular Dystrophy by Jonathan Broomfield, Keith R. Abrams, Michael J. Crowther, Michela Guglieri, Nicholas R Latimer and Mark J. Rutherford in MDM Policy & Practice
