# Supplementary material for: Evaluating the Impact of Different Natural History Modeling Methods on Cost-Effectiveness Decisions: A Case Study in Duchenne Muscular Dystrophy
Source: MDM Policy Pract. 2026 Jun 11;11(1):23814683261447231. doi: 10.1177/23814683261447231 (PMC13260772; doi:10.1177/23814683261447231)
Supplement: sj-docx-1-mpp-10.1177_23814683261447231 – Supplemental material for Evaluating the Impact of Different Natural History Modeling Methods on Cost-Effectiveness Decisions: A Case Study in Duchenne Muscular Dystrophy [file sj-docx-1-mpp-10.1177_23814683261447231.docx]

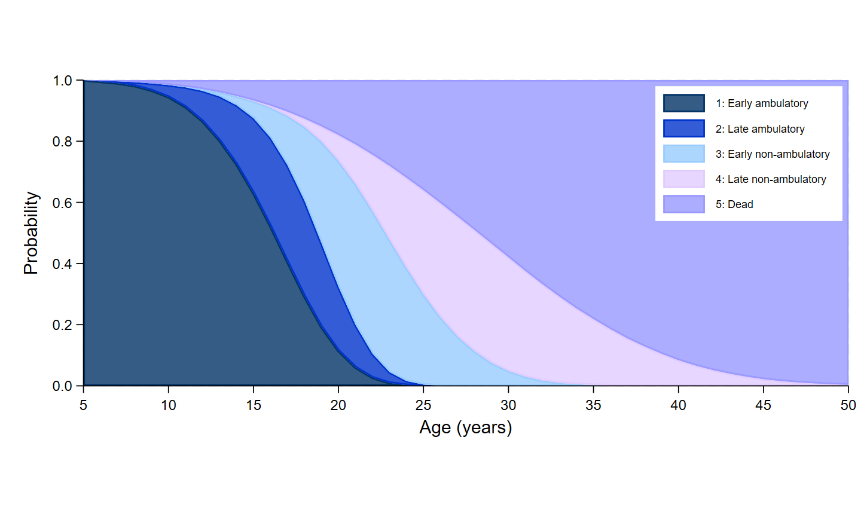

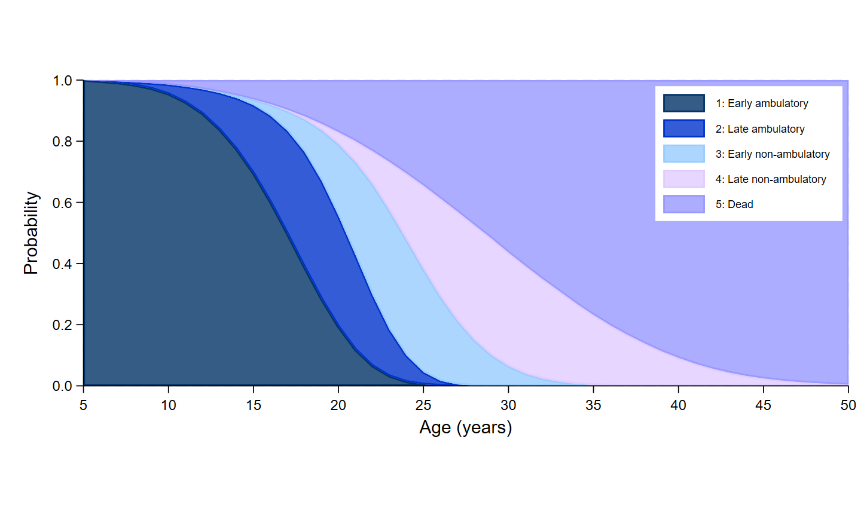

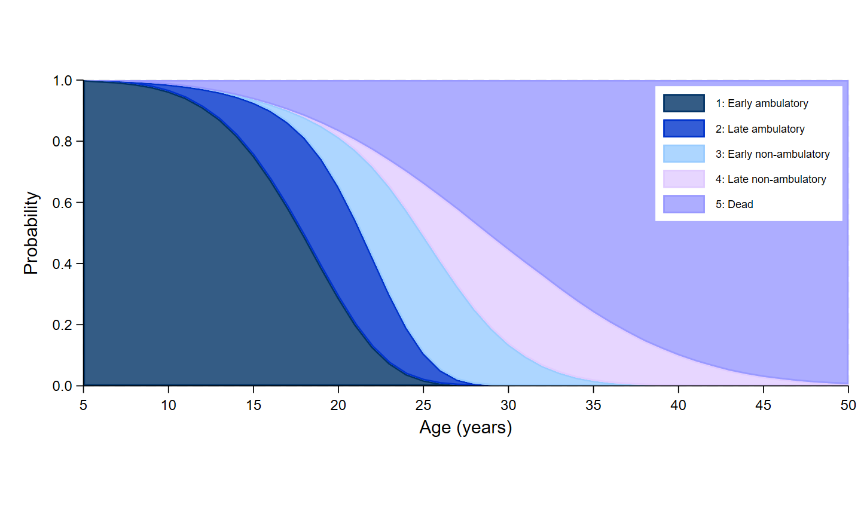

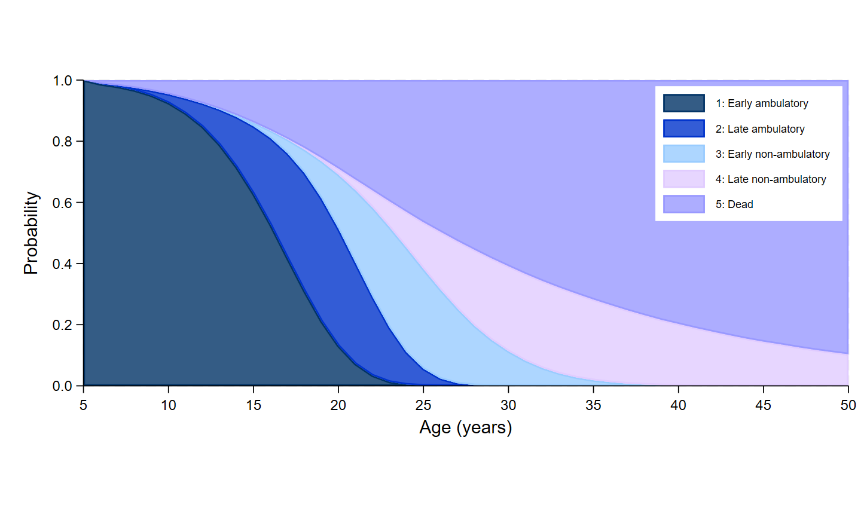

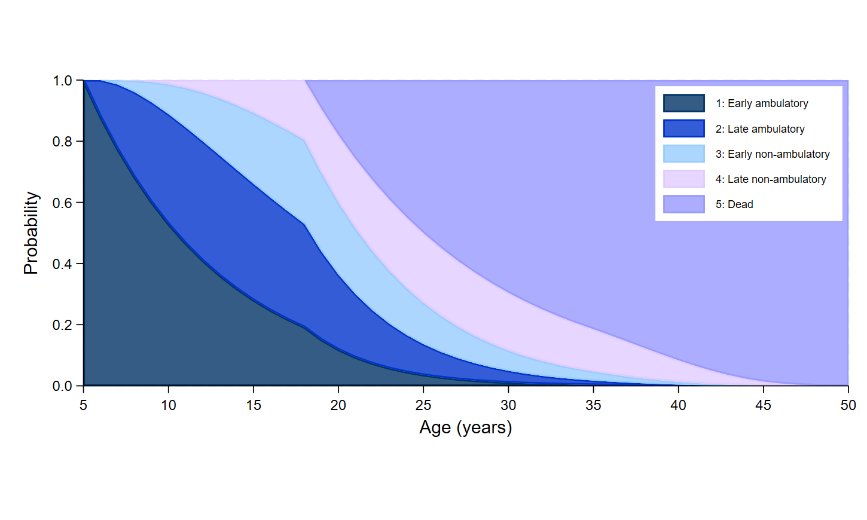


a) Assumption-based approach.

b) No-adjustment model.

c) One-stage frailty model.

d) Two-stage proportional model.

e) Two-stage stratified model.

Supplementary Figure 1: Health state occupancies after age 5 across the ambulatory disease states from the assumption-based and four model-based approaches for the treatment cohort.
