# Supplementary material for: Evaluating the Impact of Different Natural History Modeling Methods on Cost-Effectiveness Decisions: A Case Study in Duchenne Muscular Dystrophy
Source: MDM Policy Pract. 2026 Jun 11;11(1):23814683261447231. doi: 10.1177/23814683261447231 (PMC13260772; doi:10.1177/23814683261447231)
Supplement: sj-docx-2-mpp-10.1177_23814683261447231 – Supplemental material for Evaluating the Impact of Different Natural History Modeling Methods on Cost-Effectiveness Decisions: A Case Study in Duchenne Muscular Dystrophy [file sj-docx-2-mpp-10.1177_23814683261447231.docx]

Supplementary materials: D-RSC datasets

Table 1: D-RSC^1^ datasets

| Study | Region | Study type | N patients | Age range (years)^†^ | Median (IQR) follow-up | Date collected | Inclusion criteria |
| --- | --- | --- | --- | --- | --- | --- | --- |
| UC Davis | USA | Natural history | 73 | 2–31 | 10.4 (7.9, 13.7) | 1980s | Unknown |
| UC Davis 2^2,3^ | USA | Test/re-test data for clinical outcome | 24 | 4–14 | 7.8 (6.1, 9.4) | Publication accepted in 2009 | Walk 10m,  no acute illness |
| CCHMC^4^ | USA | Clinical | 97 | 7–16 | 7.4 (7.2, 7.9) | Visited 2011–‍2015 | ≥3 years follow-up |
| CINRG DNHS^5,6^ | Argentina, Australia, Canada, India, Israel, Italy, Puerto Rico, Sweden, USA | Natural history | 440 | 2-30 | 8.9 (6.2, 14.0) | Recruited 2006–‍2009, then again 2012–2016 | No steroid-naïve, no ambulation aged 16 |
| Santhera^7,8^ | Austria, Belgium, France, Germany, Italy, Netherlands, Spain, Sweden, Switzerland, USA | Placebo arm of trial | 34 | 10–18 | 14.9  (12.0, 16.0) | Recruited 2009–2012 | PEF%p < 80% at baseline, no spinal surgery/ventilation |
| Lilly^9^ | Argentina, Belgium, Canada, France, Germany, Italy, Japan, Netherlands, Russia, South Korea, Spain, Taiwan, Turkey, UK, USA | Placebo arm of trial | 115 | 7–14 | 9.2 (8.0, 10.2) | Study ran 2013–2015 | 6MWD between 200 and 400m, left ventricular ejection fraction ≥50% |
| CHOP^10^ | USA | Clinical | 66 | 13-33 | 10.1 (7.5, 12.2) | Visited 2005–‍2010 | English speaking |
| Imaging DMD^11^ | USA | Natural history | 100 | 5–18 | 8.0 (6.7, 9.9) | Recruitment began in 2010 | Walk 100m, climb four stairs |
| PTC 007^12^ | Australia, Belgium, Canada, France, Germany, Italy, Israel, Spain, Sweden, UK, USA | Placebo arm of trial | 52 | 5–15 | 8.0 (7.0, 9.0) | Study ran 2008–2009 | Walk 75m, no daytime ventilation |
| PTC 020^13^ | Australia, Belgium, Brazil, Canada, Chile, Czech Republic, France, Germany, Israel, Italy, Poland, South Korea, Spain, Sweden, Switzerland, Turkey, UK, USA | Placebo arm of trial | 115 | 7–15 | 9.0 (7.9, 10.0) | Recruited 2013–2014 | Baseline 6MWD of 150m, no daytime ventilation |
| CINRG steroid^14^ | Australia, India, Israel, USA | Clinical trial of steroids | 64 | 4–12 | 7.2 (5.9, 8.4) | Study ran 2004–2007 | Ambulant, muscle weakness |

†Inclusion criteria ≥4 years of age as not all measures are appropriate below this age and there are limited data available.
Abbreviations: 6MWD, 6-minute walk distance; CHOP, Children’s Hospital of Philadelphia CINRG DNHS, the Cooperative International Neuromuscular Research Group Duchenne Natural History Study; CCMMC, Cincinnati Children’s Hospital Medical Center; DMD, Duchenne muscular dystrophy; D-RSC, Duchenne Regulatory Science Consortium; IQR, interquartile range; m, metre; PEF%p , peak expiratory flow percent predicted; PTC, PTC Therapeutics; UC Davis, University of California, Davis.

Table 1: Scope of the D-RSC dataset^1^.

1. Conrado, D.J., Larkindale, J., Berg, A., Hill, M., Burton, J., Abrams, K.R., Abresch, R.T., Bronson, A., Chapman, D., Crowther, M. and Duong, T., 2019. Towards regulatory endorsement of drug development tools to promote the application of model-informed drug development in Duchenne muscular dystrophy. Journal of pharmacokinetics and pharmacodynamics, 46(5), pp.441-455.
2. Henricson, E., Abresch, R., Han, J.J., Nicorici, A., Keller, E.G., Elfring, G., Reha, A., Barth, J. and McDonald, C.M., 2012. Percent-predicted 6-minute walk distance in duchenne muscular dystrophy to account for maturational influences. PLoS currents, 4.
3. McDonald, C.M., Henricson, E.K., Han, J.J., Abresch, R.T., Nicorici, A., Elfring, G.L., Atkinson, L., Reha, A., Hirawat, S. and Miller, L.L., 2010. The 6‐minute walk test as a new outcome measure in Duchenne muscular dystrophy. Muscle & Nerve: Official Journal of the American Association of Electrodiagnostic Medicine, 41(4), pp.500-510.
4. Wong, B.L., Rybalsky, I., Shellenbarger, K.C., Tian, C., McMahon, M.A., Rutter, M.M., Sawnani, H. and Jefferies, J.L., 2017. Long-term outcome of interdisciplinary management of patients with Duchenne muscular dystrophy receiving daily glucocorticoid treatment. The Journal of pediatrics, 182, pp.296-303.
5. McDonald, C.M., Henricson, E.K., Abresch, R.T., Han, J.J., Escolar, D.M., Florence, J.M., Duong, T., Arrieta, A., Clemens, P.R., Hoffman, E.P. and Cnaan, A., 2013. The cooperative international neuromuscular research group Duchenne natural history study—a longitudinal investigation in the era of glucocorticoid therapy: design of protocol and the methods used. Muscle & nerve, 48(1), pp.32-54.
6. McDonald, C.M., Henricson, E.K., Abresch, R.T., Duong, T., Joyce, N.C., Hu, F., Clemens, P.R., Hoffman, E.P., Cnaan, A., Gordish-Dressman, H. and Vishwanathan, V., 2018. Long-term effects of glucocorticoids on function, quality of life, and survival in patients with Duchenne muscular dystrophy: a prospective cohort study. The Lancet, 391(10119), pp.451-461.
7. Buyse, G.M., Voit, T., Schara, U., Straathof, C.S., D'Angelo, M.G., Bernert, G., Cuisset, J.M., Finkel, R.S., Goemans, N., McDonald, C.M. and Rummey, C., 2015. Efficacy of idebenone on respiratory function in patients with Duchenne muscular dystrophy not using glucocorticoids (DELOS): a double-blind randomised placebo-controlled phase 3 trial. The Lancet, 385(9979), pp.1748-1757.
8. Buyse, G.M., Voit, T., Schara, U., Straathof, C.S., D'Angelo, M.G., Bernert, G., Cuisset, J.M., Finkel, R.S., Goemans, N., Rummey, C. and Leinonen, M., 2017. Treatment effect of idebenone on inspiratory function in patients with Duchenne muscular dystrophy. Pediatric pulmonology, 52(4), pp.508-515.
9. Victor, R.G., Sweeney, H.L., Finkel, R., McDonald, C.M., Byrne, B., Eagle, M., Goemans, N., Vandenborne, K., Dubrovsky, A.L., Topaloglu, H. and Miceli, M.C., 2017. A phase 3 randomized placebo-controlled trial of tadalafil for Duchenne muscular dystrophy. Neurology, 89(17), pp.1811-1820.
10. Mayer, O.H., Finkel, R.S., Rummey, C., Benton, M.J., Glanzman, A.M., Flickinger, J., Lindström, B.M. and Meier, T., 2015. Characterization of pulmonary function in Duchenne muscular dystrophy. Pediatric pulmonology, 50(5), pp.487-494.
11. Barnard, A.M., Willcocks, R.J., Finanger, E.L., Daniels, M.J., Triplett, W.T., Rooney, W.D., Lott, D.J., Forbes, S.C., Wang, D.J., Senesac, C.R. and Harrington, A.T., 2018. Skeletal muscle magnetic resonance biomarkers correlate with function and sentinel events in Duchenne muscular dystrophy. PLoS One, 13(3), p.e0194283.
12. Bushby, K., Finkel, R., Wong, B., Barohn, R., Campbell, C., Comi, G.P., Connolly, A.M., Day, J.W., Flanigan, K.M., Goemans, N. and Jones, K.J., 2014. Ataluren treatment of patients with nonsense mutation dystrophinopathy. Muscle & nerve, 50(4), pp.477-487.
13. McDonald, C.M., Campbell, C., Torricelli, R.E., Finkel, R.S., Flanigan, K.M., Goemans, N., Heydemann, P., Kaminska, A., Kirschner, J., Muntoni, F. and Osorio, A.N., 2017. Ataluren in patients with nonsense mutation Duchenne muscular dystrophy (ACT DMD): a multicentre, randomised, double-blind, placebo-controlled, phase 3 trial. The Lancet, 390(10101), pp.1489-1498.
14. Escolar, D.M., Hache, L.P., Clemens, P.R., Cnaan, A., McDonald, C.M., Viswanathan, V., Kornberg, A.J., Bertorini, T.E., Nevo, Y., Lotze, T. and Pestronk, A., 2011. Randomized, blinded trial of weekend vs daily prednisone in Duchenne muscular dystrophy. Neurology, 77(5), pp.444-452.
