# Supplementary material for: Evaluating the Impact of Different Natural History Modeling Methods on Cost-Effectiveness Decisions: A Case Study in Duchenne Muscular Dystrophy
Source: MDM Policy Pract. 2026 Jun 11;11(1):23814683261447231. doi: 10.1177/23814683261447231 (PMC13260772; doi:10.1177/23814683261447231)
Supplement: sj-docx-3-mpp-10.1177_23814683261447231 – Supplemental material for Evaluating the Impact of Different Natural History Modeling Methods on Cost-Effectiveness Decisions: A Case Study in Duchenne Muscular Dystrophy [file sj-docx-3-mpp-10.1177_23814683261447231.docx]

# Supplementary materials: Sensitivity analysis results


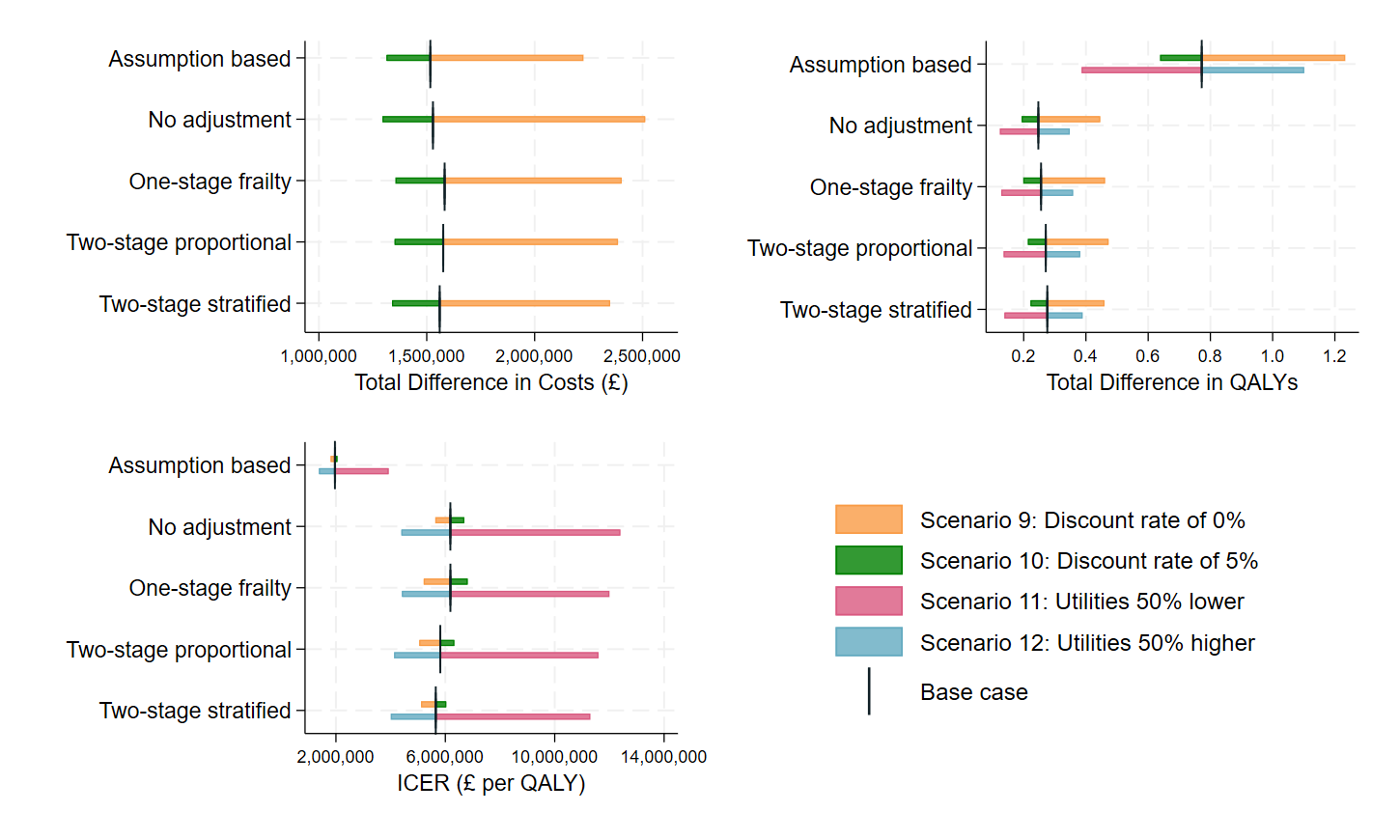
The resulting differences in lifetime costs and QALYs, and corresponding ICERs, from scenarios 9-12 are shown in Figure A1. Results from scenarios 13 and 14, where baseline medical costs were varied, are not plotted as they had virtually no impact on the estimated differences in costs and QALYs and ICERs and would not be visible on the plot.

Figure A1: Total difference in costs and QALYs and corresponding ICERs obtained from the five methods from the remaining scenarios in the sensitivity analysis of the discount rate, utilities and baseline costs.

Table A1: Total differences in costs and QALYs and corresponding ICERs obtained from the five methods in the base case analysis and first 8 scenarios from the sensitivity analysis.

| **Estimate** | **Method** | **Base case** | **Scenario 1** | **Scenario 2** | **Scenario 3** | **Scenario 4** | **Scenario 5** | **Scenario 6** | **Scenario 7** | **Scenario 8** |
| --- | --- | --- | --- | --- | --- | --- | --- | --- | --- | --- |
| ∆ Costs (£) | Assumption-based | £1,520,000 | £1,630,000 | £1,000,000 | £630,000 | £470,000 | £327,000 | £1,490,000 | £1,750,000 | £1,620,000 |
|  | No-adjustment | £1,530,000 | £1,710,000 | £1,130,000 | £960,000 | £260,000 | £200,000 | £1,530,000 | £1,970,000 | £1,730,000 |
|  | One-stage frailty | £1,580,000 | £1,690,000 | £1,230,000 | £1,060,000 | £240,000 | £210,000 | £1,570,000 | £1,840,000 | £1,700,000 |
|  | Two-stage proportional | £1,580,000 | £1,690,000 | £1,180,000 | £1,000,000 | £240,000 | £210,000 | £1,560,000 | £1,830,000 | £1,680,000 |
|  | Two-stage stratified | £1,560,000 | £1,670,000 | £1,090,000 | £950,000 | £190,000 | £260,000 | £1,550,000 | £1,820,000 | £1,660,000 |
| ∆ QALYs | Assumption-based | 0.77 | 1.01 | 0.55 | 0.50 | 0.42 | 0.08 | 1.71 | 2.49 | 2.03 |
|  | No-adjustment | 0.25 | 0.62 | 0.15 | 0.32 | 0.21 | 0.06 | 0.54 | 1.53 | 1.09 |
|  | One-stage frailty | 0.26 | 0.48 | 0.17 | 0.25 | 0.16 | 0.06 | 0.58 | 1.18 | 0.88 |
|  | Two-stage proportional | 0.27 | 0.48 | 0.17 | 0.23 | 0.17 | 0.05 | 0.60 | 1.17 | 0.87 |
|  | Two-stage stratified | 0.28 | 0.48 | 0.19 | 0.25 | 0.13 | 0.06 | 0.65 | 1.17 | 0.85 |
| ICER (£ per QALY) | Assumption-based | £1,960,000 | £1,610,000 | £1,800,000 | £1,270,000 | £1,120,000 | £3,890,000 | £870,000 | £700,000 | £800,000 |
|  | No-adjustment | £6,180,000 | £2,730,000 | £7,740,000 | £3,010,000 | £1,260,000 | £3,170,000 | £2,830,000 | £1,290,000 | £1,600,000 |
|  | One-stage frailty | £6,190,000 | £3,500,000 | £7,140,000 | £4,250,000 | £1,490,000 | £3,460,000 | £2,710,000 | £1,570,000 | £1,920,000 |
|  | Two-stage proportional | £5,820,000 | £3,480,000 | £6,810,000 | £4,341,000 | £1,460,000 | £4,490,000 | £2,600,000 | £1,570,000 | £1,940,000 |
|  | Two-stage stratified | £5,650,000 | £3,470,000 | £5,750,000 | £3,850,000 | £1,470,000 | £4,360,000 | £2,370,000 | £1,550,000 | £1,950,000 |

Base case: 25% reduction in all intermediate transition rates.
Scenario 1: 25% reduction in all mortality as well as intermediate transition rates.
Scenario 2: 25% and 10% reduction in transition rates between states 1 and 2 and states 2 and 3 respectively (diminishing treatment effect.
Scenario 3: 25% reduction in both transition rates out of state 1 (treatment targeted at early ambulatory patients).
Scenario 4: 25% reduction in both transition rates out of state 2 (treatment targeted at late ambulatory patients).
Scenario 5: 25% reduction in both transition rates out of state 3 (treatment targeted at early non-ambulatory patients).
Scenario 6: 50% reduction in all intermediate transition rates (greater treatment effect).
Scenario 7: 50% reduction in all intermediate and mortality transition rates (even greater treatment effect).
Scenario 8: 50%, 40% and 20% reduction in all intermediate and mortality transition rates out of states 1, 2 and 3 respectively (greater but diminishing treatment effect).


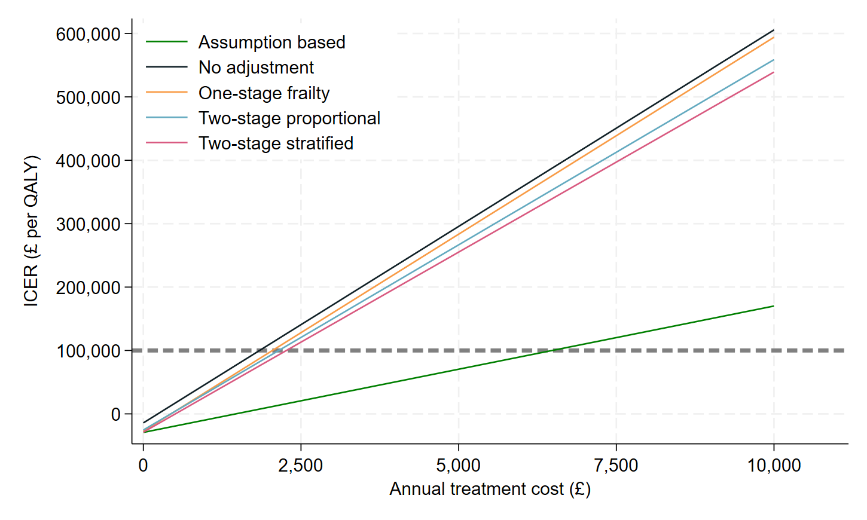


b) Annual difference in costs.


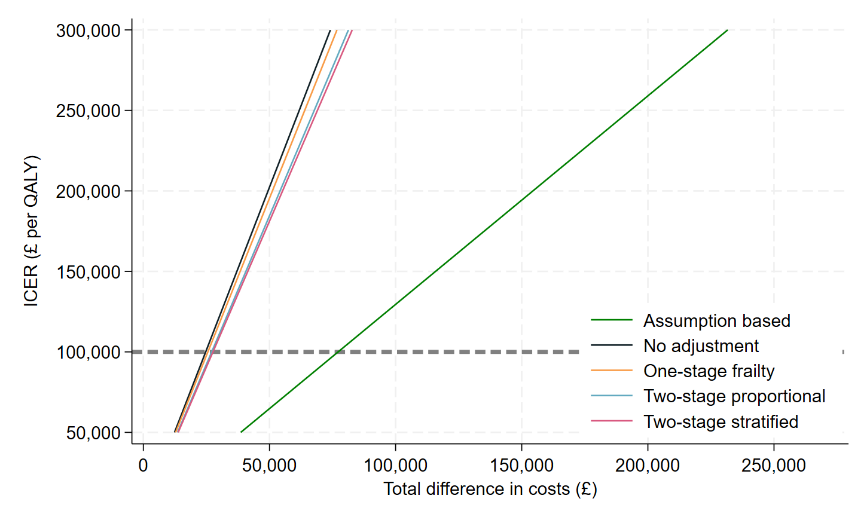


a) Total difference in costs.


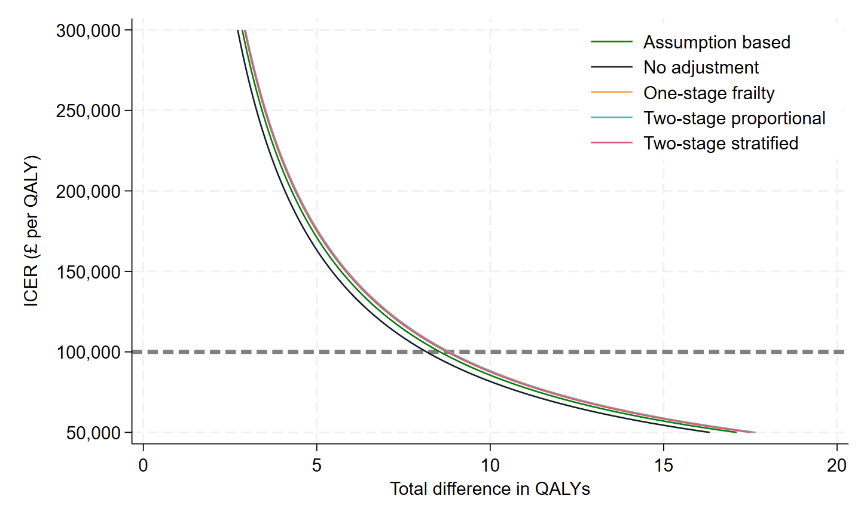


c) Total difference in QALYs.

Figure A2: Threshold analysis on the total difference in costs, annual difference in costs and total difference in QALYs required to obtain an ICER of £100,000 per QALY
