# Supplementary material for: Evaluating the Impact of Different Natural History Modeling Methods on Cost-Effectiveness Decisions: A Case Study in Duchenne Muscular Dystrophy
Source: MDM Policy Pract. 2026 Jun 11;11(1):23814683261447231. doi: 10.1177/23814683261447231 (PMC13260772; doi:10.1177/23814683261447231)
Supplement: sj-docx-4-mpp-10.1177_23814683261447231 – Supplemental material for Evaluating the Impact of Different Natural History Modeling Methods on Cost-Effectiveness Decisions: A Case Study in Duchenne Muscular Dystrophy [file sj-docx-4-mpp-10.1177_23814683261447231.docx]

**Supplementary materials: Team members from participating CINRG sites,** the Duchenne Regulatory Science Consortium (D-RSC), and the ImagingDMD network

| **CINRG sites** | **Team members** |
| --- | --- |
| University of California Davis | C. McDonald, E. Henricson, M. Cregan, L. Johnson, J. Han, N. Joyce, A. Nicorici, D. Reddy |
| Sundaram Medical Foundation and Apollo Children’s Hospital, Alberta Children’s Hospital, Calgary | J. Mah, A. Chiu, T. Haig, M. Harris, M. Kornelsen, N. Rincon, K. Sanchez, L. Walker |
| Queen Silvia Children’s Hospital | M. Tulinius, A. Alhander, A. Ekstrom, A. Gustafsson, A. Kroksmark, U. Sterky, L. Wahlgren |
| Children’s National Health System | R. Leshner, N. Brody, B. Drogo, M. Leach, C. Tesi-Rocha, M. Birkmeier, B. Tadese, A. Toles, M. Thangarajh |
| Royal Children’s Hospital | A. Kornberg, K. Carroll, K. DeValle, R. Kennedy, V. Rodriguez, D. Villano |
| Hadassah Hebrew University Hospital | Y. Nevo, R. Adani, A. Bar Leve, L. Chen-Joseph, M. Daana, V. Panteleyev-Yitshak, E. Simchovitz, D. Yaffe |
| Instituto de Neurosciencias Fundacion Favaloro | L. Andreone, F. Bonaudo, J. Corderi, L. Levi, L. Mesa, P. Marco |
| Children’s Hospital of Pittsburgh of UPMC and the University of Pittsburgh | P. Clemens, H. Abdel-Hamid, R. Bendixen, C. Bise, A. Craig, K. Karnavas, C. Matthews, G. Niizawa, A. Smith, J. Weimer |
| Washington University | J. Anger, T. Christenson, J. Florence, R. Gadeken, P. Golumbak, B. Malkus, A. Pestronk, R. Renna, J. Schierbecker, C. Seiner, C. Wulf |
| Children’s Hospital of Richmond at VCU | J. Teasley, S. Blair, B. Grillo, E. Monasterio |
| University of Tennessee | T. Bertorini, M. Barrett-Adair, C. Benzel, K. Carter, J. Clift, B. Gatlin, R. Henegar, J. Holloway, M. Igarashi, F. Kiphut, A. Parker, A. Phillips, R. Young |
| Children’s Hospital of Westmead | K. North, K. Cornett, N. Gabriel, M. Harman, C. Miller, K. Rose, S. Wicks |
| University of Alberta | H. Kolski, L. Chen, C. Kennedy; Centro Clinico Nemo: M. Beneggi, L. Capone, A. Molteni, V. Morettini |
| Texas Children’s Hospital | T. Lotze, A. Gupta, A. Knight, B. Lott, R. McNeil, G. Orozco, R. Schlosser |
| University of Minnesota | G. Chambers, J. Day, J. Dalton, A. Erickson, M. Margolis, J. Marsh, C. Naughton |
| Mayo Clinic | K. Coleman-Wood, A. Hoffman, W. Korn-Petersen, N. Kuntz |
| University of Puerto Rico | B. Deliz, S. Espada, P. Fuste, C. Luciano, J. Torres |
| CINRG Coordinating Center | L. Morgenroth, M. Ahmed, A. Arrieta, N. Bartley, T. Brown-Caines, C. Carty, T. Duong, J. Feng, F. Hu, L. Hunegs, Z. Sund, W. Tang, A. Zimmerman |

Abbreviations: CINRG, Cooperative International Neuromuscular Research Group; UPMC, University of Pittsburgh Medical Center; VCU, Virginia Commonwealth University.

The Cooperative International Neuromuscular Research Group Duchenne Natural History Study (CINRG-DNHS) was funded by:

- The U.S. Department of Education/National Institute on Disability and Rehabilitation Research (NIDRR) (#H133B031118, #H133B090001)
- The U.S. Department of Defense (#W81XWH-12-1-0417)
- The National Institutes of Health/ National Institute of Arthritis and Musculoskeletal and Skin Diseases (NIAMS) (#R01AR061875)
- The Parent Project Muscular Dystrophy.

The Duchenne Regulatory Science Consortium (D-RSC) includes: Binghamton University, Children’s Hospital of Philadelphia, Children's National Health System, Children’s National Heart Institute, Cincinnati Children’s Hospital Medical Center, Hadassah Medical Center, Indiana University School of Medicine, Leiden University Medical Center, Nationwide Children’s Hospital, Oregon Health & Science University, Stanford University, University of California Davis, UMass Memorial, University of Arizona, University of Florida, University of Leicester, UK, Vanderbilt University Medical Center , Parent Project Muscular Dystrophy, CureDuchenne, Astellas, Avidity Biosciences, Edgewise Therapeutics, Entrada Therapeutics, NS Pharma, Pfizer, REGENXBIO, Roche, Sarepta Therapeutics, Takeda , Ultragenyx, Vertex. Funding in support of D-RSC was made possible, in part, by the U.S. Food and Drug Administration (FDA) through the grant 5U18 FD 005320.

With special thanks to the ImagingDMD network Investigators from the University of Florida (UF), the Children's Hospital of Philadelphia (CHOP), Oregon Health and Science University (OHSU), and Shriners Hospital for Children-Portland. ImagingDMD’s data was supported by NIH grant R01AR056973.
